# The relationship between different facets of empathy, pain perception and compassion fatigue among physicians

**DOI:** 10.3389/fnbeh.2014.00243

**Published:** 2014-07-11

**Authors:** Ezequiel Gleichgerrcht, Jean Decety

**Affiliations:** ^1^Department of Medicine and Department of Psychology, Favaloro UniversityBuenos Aires, Argentina; ^2^UDP-INECO Foundation Core on Neuroscience (UIFCoN), Diego Portales UniversitySantiago, Chile; ^3^Department of Psychology and Department of Psychiatry and Behavioral Neuroscience, The University of ChicagoChicago, IL, USA

**Keywords:** clinical empathy, pain, compassion fatigue, burnout, medicine, gender differences

## Abstract

**Background:** Medical practitioners such as physicians are continuously exposed to the suffering and the distress of patients. Understanding the way pain perception relates to empathetic dispositions and professional quality of life can contribute to the development of strategies aimed at protecting health professionals from burnout and compassion fatigue. In the present study we investigate the way individual dispositions relate to behavioral measures of pain sensitivity, empathy, and professional quality of life.

**Methods:** A secure Web-based series of self-report measures and a behavioral task were administered to 1,199 board-certified physicians. Additionally, surveys were used to obtain measures of demographic and professional background; dispositional empathy (empathic concern, personal distress, and perspective taking); positive (compassion satisfaction) and negative (burnout and secondary traumatic stress) aspects of their professional life. In the behavioral task, participants were asked to watch a series of video clips of patients experiencing different levels of pain and provide ratings of pain intensity and induced personal distress.

**Results:** Perceived pain intensity was significantly lower among more experienced physicians but similar across specialty fields with varying demands of emotional stress. Watching videos of patients in pain, however, elicited more personal distress among physicians in highly demanding medical fields, despite comparable empathy dispositions with other fields. The pain of male patients was perceived as less intense than the pain of female patients, and this effect was more marked for female physicians. The effect of dispositional empathy on pain perception and induced personal distress was different for each sub-component, with perspective taking and empathic concern (EC) being predictive of the behavioral outcomes. Physicians who experience both compassion satisfaction and fatigue perceive more pain and suffer more personal distress from it than those who only suffer the negative aspects of professional quality of life.

**Conclusions:** Professional experience seems to desensitize physicians to the pain of others without necessarily helping them down-regulate their own personal distress. Pain perception is also related with specific aspects of empathy and varies depending on context, as is the case with the gender of their patients. Minimum levels of empathy appear necessary to benefit from the positive aspects of professional quality of life in medicine.

## Introduction

Clinical empathy is an essential element of quality care, associated with improved patient satisfaction, increased adherence to treatment, and fewer malpractice complaints (Burns and Nolen-Hoeksema, 1992; Rakel et al., 2009, 2011; Hojat et al., 2011; Del Canale et al., 2012) as well as increased physician health, well-being, and professional satisfaction (Mercer and Reynolds, 2002; Benbassat and Baumal, 2004). However, despite all these clear advantages to both patients and physicians, empathy in medicine remains an undervalued and understudied topic (Riess, 2010; Schattner, 2012). Maintaining appropriate levels of clinical empathy is challenging because medical practitioners routinely deal with the most emotionally distressing situations—illness, dying, suffering in every form—and such situations can rapidly make an empathic person anxious, perhaps too anxious to be helpful (Neumann et al., 2011; Halpern, 2012). This painful reality may take its toll on these individuals and can lead to compassion fatigue, burnout, professional distress, and result in a low sense of accomplishment and severe emotional exhaustion (Figley, 2012; Gleichgerrcht and Decety, 2013). Thus, physicians may experience difficulty empathizing with their patients (Feighny et al., 1995; Bonvicini et al., 2009). For instance, a study which coded interviews between physicians and lung cancer patients found that, out of 384 empathic opportunities—defined as patients’ statements including an explicit description of emotion or patients’ statements or clue that indicated an underlying emotion—physicians responded empathically to only 39 of them (10%), most often responding with little emotional support and shifting to biomedical questions and statements instead (Morse et al., 2008). Although the reasons for such difficulties are likely complex and multifaceted, one possible explanation may be that physicians lack the cognitive and emotional resources to engage in empathic processing. The vast majority of doctors have the capacity for empathy (Handford et al., 2013). However, particular skills such as attention, self-regulation, and emotional awareness are needed to reliably respond empathically to distressed patients while making difficult decisions and performing potentially high-risk, high-demand interventions. Added to this are organizational demands for ever increasing caseloads, which reduce the time doctors can spend with patients or use to manage their own emotions. Maintaining person-oriented connections during such high stress conditions requires a great deal of attention and self-regulation, which taxes a limited supply of cognitive and emotional resources (Feighny et al., 1995; Haque and Waytz, 2012).

Empathy is a natural socio-emotional competency that has evolved with the mammalian brain to form and maintain social bonds, facilitate the survival of offspring, and facilitate cooperation among group members (Decety et al., 2012). Recently, work from developmental and affective and social neuroscience converge to consider empathy as a multidimensional construct comprising dissociable components that interact and operate in parallel fashion, including affective, motivational and cognitive components (Decety and Jackson, 2004; Decety and Svetlova, 2012). Affective sharing (also known as emotional resonance), the first element of empathy to appear during ontogeny, refers to the unconscious sharing of the affective state of another, and is independent of perspective-taking and higher levels of cognitive understanding (Decety, 2010). Empathic understanding entails the conscious awareness of the emotional state of another person, usually facilitated by perspective taking. Empathic concern (EC) refers to a motivation to care for someone in need. Successful emotion regulation enables the control of emotion, drive, and motivation in the service of adaptive behavior. Even though these components are intertwined and not completely independent of one another, it is helpful to consider them separately, as each contributes to various aspects of the experience of empathy, and possible health outcomes for the physician (Decety, 2011).

The biological predisposition to resonate emotionally with another person is regarded as a critical aspect of social interaction (van Baaren et al., 2009). This is the case when perceiving other people in pain or emotional distress, a situation common to medical practitioners. Importantly, pain evolved protective functions not only by warning the suffering person, but also by impelling expressive behaviors that attract the attention of others (Craig, 2004; Decety, 2009). Recent findings from studies in affective neuroscience revealed shared neural representations for own pain and other’s pain. Brain regions involved in the experience of physical pain including the anterior cingulate cortex (ACC), insula, periaqueductal gray, somatosensory cortex, orbitofrontal cortex, and amygdala are also activated by the perception or even the imagination of another individual in pain (Eisenberger, 2011; Lamm et al., 2011). Importantly, this affective resonance is not automatic and modulated by contextual, cognitive, social, and interpersonal variables (Coll et al., 2012; Echols and Correll, 2012). Activity in the pain neural network is significantly enhanced when individuals view or imagine their loved-ones in pain compared to strangers (Cheng et al., 2010). Affective sharing is moderated by *a priori* implicit attitudes toward conspecifics. For example, study participants were significantly more sensitive to the pain of individuals who had contracted AIDS as the result of a blood transfusion as compared to individuals who had contracted AIDS as the results of their illicit drug addiction (sharing needles), as evidenced by higher pain sensitivity ratings and greater hemodynamic activity in the ACC, insula, and periaqueductal gray (PAG), although the intensity of pain on the facial expressions was strictly the same across all videos (Decety et al., 2010a). In the context of medicine, some neuroimaging studies have reported that physicians tend to down-regulate their empathic response to the pain of others (Cheng et al., 2007; Decety et al., 2010b).

One of the consequences of sharing the pain of others is that it can lead to emotional distress in the observer. One recent study with practicing physicians demonstrated that emotional distress is strongly coupled with compassion fatigue, which, in turn, is not associated with EC and caring for others (Gleichgerrcht and Decety, 2013). It is thus possible that physicians who are most vulnerable to professional distress, which may lead to emotional exhaustion, detachment and a low sense of accomplishment, are those who have difficulties regulating their negative emotions elicited by perceiving their patients’ distress.

Can empathy, and to what extent does it need to be regulated for the sake of both patients and physicians in medical contexts? If we consider that the costs of emotional empathy among health professionals are related to high levels of emotional arousal when perceiving the distress of others, regulation of EC must be achieved by reducing it.

In this study, we examined the relationships between sensitivity to the pain of others with a number of dispositional measures of several components of empathy including perspective taking and personal distress in a large number of practicing physicians. Initially, we investigated how age, gender, experience, and medical specialty modulate pain perception and the personal distress that comes from seeing others suffering. We then sought to understand how dispositional measures of empathy relate to behavioral measures of self- and other-oriented emotional responding to pain. Finally, we explored possible associations between these behavioral outcomes and compassion satisfaction and fatigue.

## Methods

The study was approved by the Ethics Committee at the Institute of Cognitive Neurology (INECO, Buenos Aires, Argentina).

### Participants

Participants were 1,199 physicians (50.5% male, 46.5 ± 11.8 years old with 19.7 ± 12.0 years of experience as physicians, on average) who had previously completed measures for a study on empathy and burnout (Gleichgerrcht and Decety, 2013). All participants were board-certified in the countries (South American continent) were they were currently working. Participants gave their consent to participate in the study by clicking on an “I agree” button placed beneath an informed consent and explanatory letter.

### Procedure

Participants initially completed the following series of questionnaires:

#### Demographic and professional background

Participants provided information about (a) age; (b) gender; (c) field of practice; and (d) number of years working as physicians (Table 1).

**Table 1:** **Classification of medical specialties based on the opinion of 137 independent raters (who were medical doctors) assessing whether each field poses settings of high, average, or low emotional exhaustion**.

**High**	Emergentology
	Oncology
	Pediatrics
	Psychiatry
	Surgery
	Toxicology
	Traumatology
**Average**	Anesthesiology
	Cardiology
	Internal Medicine
	Gastroenterology
	Gerontology
	Infections diseases
	Nephrology
	Neurology
	Obstetrics and Gynecology
	Pulmonology
**Low**	Allergy
	Dermatology
	Diagnostic Imaging
	Ear, Nose and Throat
	Endocrinology
	Family Medicine / General Practitioners
	Genetics
	Hematology
	Immunoloy
	Legal Medicine
	Nutrition
	Ophthalmology
	Pathology
	Pharmacology
	Public Health

#### Empathy

Participants completed the Interpersonal Reactivity Inventory (IRI; Davis, 1983), which includes several 7-item subscales, assessing specific aspects of empathy, namely: Empathic Concern (EC; the tendency to experience feelings of warmth, compassion, and concern for other people), Personal Distress (PD; one’s own feelings of personal unease and discomfort in reaction to the emotions of others), and Perspective Taking (PT; the tendency to adopt the point of view of other people). EC and PD are considered two independent measures of emotional empathy focusing on the self- and other- oriented set of feelings elicited by an agent. PT, instead, is a measure of the cognitive aspect of empathy. The Fantasy subscale was excluded, as it has been designed to tap on respondent’s tendencies to transpose themselves imaginatively into the feelings and actions of fictitious characters in books, movies, and plays, an ability that is not directly relevant for the purposes of the present study.

#### Professional quality of life

Positive and negative aspects were assessed with the Professional Quality of Life Scale V (ProQOL) developed by Stamm (2008). This scale comprises 30 items rated on a 1 (never) to 5 (very often) scale to obtain three measures: (a) compassion satisfaction (CS), or the pleasure derived from being able to perform one’s job well; (b) burnout (BO), one of the elements of compassion fatigue, particularly associated with feelings of hopelessness and difficulties in dealing with work or in performing one’s job effectively; and (c) Secondary Traumatic Stress (STS), which is another component of compassion fatigue, consisting of work-related secondary exposure to extreme or traumatic stressful events. Following cut-off scores established by the original ProQoL manual, participants were classified into low, average, and high groups for each subdomain as follows: lo-CS ≤ 44; 44 < avg-CS < 57; hi-CS ≥ 57; lo-BO ≤ 43; 43 < avg-CS < 56; hi-CS ≥ 56; lo-STS ≤ 42; 42 < avg-CS < 56; hi-CS ≥ 56.

Participants then viewed 12 video clips showing individuals (6 males and 6 females) expressing physical pain, in a random order. Video clips used in the behavioral task have been validated for valence and intensity and used in previous behavioral and functional neuroimaging studies (Lamm et al., 2007; Decety et al., 2010a). These clips were shot from a frontal view using a high-resolution digital color camcorder, were centered on the target’s nose, and showed the whole head and parts of the shoulders. Videos were taken against a light blue background curtain (as used in hospitals), and targets were wearing a white medical blouse in order to recreate the setting of a hospital. Videos included in the present study all showed a natural pain response in which targets displayed brow lowering, orbit tightening, and either cursing/pressing of the lips or opening/stretching of the mouth, a stereotyped motor pattern previously attributed to the facial expression of pain (Craig and Patrick, 1985). We included, in random order, two videos of people experiencing happiness. These videos were used to control for random answers, as participants included in the present study were all below the 10th percentile of perceived pain intensity and induced personal distress of both happy videos, thus ensuring that they were paying attention to the stimuli presented to them.

### Statistical analysis

Comparisons of dependent variables between two groups at a time (e.g., female vs. male participants, more vs. less experienced physicians, etc.) were conducted. Student’s *t* test and one-way ANOVA was used to compare variables across three or more groups at a time. In addition, ANCOVAs were carried out to control for the effect of potential confounding variables. For instances in which the main effect of given factors and their interactions became relevant (e.g., compassion fatigue and compassion satisfaction), a factorial 2 × 2 ANOVA design was employed. When analyzing categorical variables (e.g., gender), the Fisher exact probability test for contingency tables was used. Correlations between variables were analyzed using Pearson’s correlation coefficient. The α value for all statistical tests was set at 0.05, two-tailed (Tables 2 and 3).

**Table 2:** **Comparison of demographic, IRI and ProQoL variables between participants group based on their type of specialty**.

		**Low**	**Average**	**High**	**Statistical Test**
**Age**		**44.0 (12.5)**	**48.5 (11.4)**	**48.3 (10.5)**	***F*_(2,1196)_ = 20.4, *p* < 0.001**
**Gender (M : F)**		**253 : 245**	**187 : 188**	**166 : 160**	***X*^2^ = 0.1, *p* = 0.95**
**Years of experience**		**17.1 (12.4)**	**21.7 (11.6)**	**21.5 (11.3)**	***F*_(2,1196)_ = 20.7, *p* < 0.001**
IRI	EC	31.2 (5.1)	31.2 (5.0)	31.4 (5.2)	*F*_(2,1196)_ = 0.20, *p* = 0.79
	PD	13.3 (4.4)	13.1 (4.5)	13.1 (4.4)	*F*_(2,1196)_ = 1.65, *p* = 0.20
	PT	24.1 (4.6)	24.2 (4.9)	23.9 (4.9)	*F*_(2,1196)_ = 0.36, *p* = 0.70
ProQoL	CS	49.1 (6.7)	48.3 (7.2)	49.1 (6.7)	*F*_(2,1196)_ = 1.92, *p* = 0.15
	BO	29.3 (6.6)	29.8 (6.3)	30.0 (7.2)	*F*_(2,1196)_ = 1.30, *p* = 0.27
	STS	25.8 (7.4)	25.9 (7.3)	26.7 (7.4)	*F*_(2,1196)_ = 1.28, *p* = 0.28

*Values are *Mean* (*SD*). IRI = Interpersonal Reactivity Index, EC = Empathic Concern, PD = Personal Distress, PT = Perspective Taking, ProQoL = Professional Quality of Life, CS = Compassion Satisfaction, BO = Burnout, STS = Secondary Traumatic Stress*.

**Table 3:** **Summary of correlations found between subjective ratings of pain intensity and induced personal distress and individual dispositions for the different facets of empathy and other variables**.

	**Perceived Pain Intensity**	**Induced Personal Distress**	**Age**	**Years of Experience**	**Perspective Taking**	**Empathic Concern**	**Personal Distress**	**Compassion Satisfaction**	**Burnout**	**Secondary Traumatic Stress**
Perceived Pain Intensity	–	*R* = 0.62 *P* < 0.001	*R* = −0.14 *P* = 0.04	*R* = −0.15 *P* = 0.03	*R* = 0.11 *P* < 0.001	*R* = 0.03 *P* = 0.29	*R* = 0.024 *P* = 0.45	*R* = 0.02 *P* = 0.57	*R* = 0.025 *P* = 0.40	*R* = −0.002 *P* = 0.94
Induced Personal Distress	*R* = 0.62 *P* < 0.001	–	*R* = −0.02 *P* = 0.35	*R* = −0.01 *P* = 0.67	*R* = 0.07 *P* = 0.01	*R* = 0.08 *P* = 0.01	*R* = 0.04 *P* = 0.28	*R* = 0.049 *P* = 0.10	*R* = 0.02 *P* = 0.49	*R* = 0.038 *P* = 0.20

*Shadowed cells highlight significant correlations*.

## Results

### Effects of age, professional experience, and specialty

**Figure 1:**
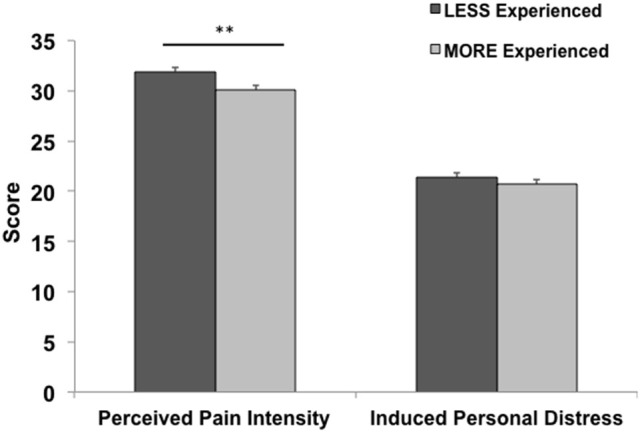
**The effect of professional medical experience**. Mean (*S.E.M.*) for behavioral measures in less vs. more experienced physicians. More experienced physicians perceived significantly lower rates of pain (**p < 0.01). Error bars are *S.E.M.*

A significant negative correlation was found between perceived pain intensity and both age (*r* = −0.14, *p* = 0.04) and years of experience (*r* = −0.15, *p* = 0.03). See Table 3 for a complete list of correlations with behavioral measures. Participants grouped into either a less or a more experienced group based on whether their individual years of medical practice were above (*n* = 580) or below (*n =* 619) the sample’s mean also differed on the intensity of pain perceived (*t*_1197_ = 2.79, *p* = 0.005, Cohen’s *d* = 0.16). This main effect remained (*F*_1,1196_ = 7.57, *p* = 0.006, Cohen’s *d* = 0.15) even after controlling for the effect of age, which differed significantly (*t*_1197_ = −38.6, *p* < 0.001, Cohen’s *d* = 2.23) between more (55.9 ± 8.5) and less (35.7 ± 8.2) experienced physicians (Figure 1). No significant correlations or main effects were found for induced personal distress.

One hundred and fifty physicians were randomly selected from the pool of participants to complete an addition independent task. We initially compiled a list containing all fields of practice of participants in this study. We presented this list and asked them to classify each field of medical practice depending on whether it continuously posed a setting of high, average or low emotional exhaustion based on their personal opinion. Each participant could choose only one of the three classifications for each field; 91.3% raters completed this task in its entirety. Specialties were thus classified as shown in Table 1 based on the group (high, average or low) that obtained the most votes. Participants were thus grouped into high (*n* = 326), average (*n* = 375) or low (*n* = 498) emotionally demanding specialty groups. Significant differences between these groups were found for age and years of experience, but not for gender or any of the IRI and ProQoL domains (see Table 2). For this reason, age and years of experience were used as covariates when comparing behavioral measures. Accordingly, participants whose fields of medical practice were identified as continuously posing settings of high emotional exhaustion perceived pain at equivalent levels (31.1) as physicians in fields posing average (30.7) or low (31.2) settings of emotional exhaustion (*F*_2,1193_ = 0.24, *p* = 0.79). However, there was a significant difference for the personal distress induced by the stimuli (*F*_2,1193_ = 3.81, *p* = 0.02), with highly demanding fields eliciting more personal stress (22.4) than average (20.1) or low (21.0) demanding fields. No significant differences were found, however, on any of the dispositional measures of empathy between these groups (Table 2).

### The effect of gender

**Figure 2:**
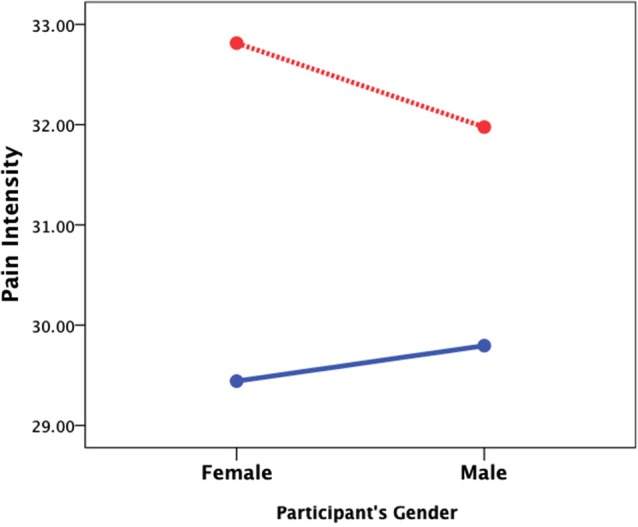
**The effect of gender**. A 2 (target’s gender) × 2 (perceiver’s gender) ANCOVA controlling for age and years of medical experience revealed that female patients (red line) are usually perceived as experiencing more severe pain and that female physicians perceive stronger pain when the patient is also a female than when it is a male patient (blue line).

Women in the sample were significantly younger than men (43.8 ± 11.2 vs. 50.1 ± 12.9; *t*_1197_ = −8.97, *p* < 0.001, Cohen’s *d* = 0.52) and had less years of experience working as physicians (16.5 ± 10.9 vs. 22.8 ± 22.8; *t*_1197_ = −9.36, *p* < 0.001, Cohen’s *d* = 0.54). The effect of gender was thus studied both for the target and the physician (perceiver) with a 2 (gender of the target) × 2 (gender of the perceiver) ANCOVA controlling for age and years of experience. For perceived pain intensity, there was a main effect of target’s gender (*F*_1,1195_ = 8.62, *p* < 0.01, Cohen’s *d* = 0.17) and a significant interaction of the target’s and perceiver’s gender (*F*_1,1195_ = 5.63, *p* < 0.01, Cohen’s *d* = 0.14) such that female physicians reported higher ratings of pain intensity when the target was female than male (Figure 2). For induced personal distress, no main effects or interactions were found (target’s gender: *F*_1,1195_ = 2.95, *p* = 0.09, Cohen’s *d* = 0.06; perceiver’s gender: *F*_1,1195_ = 0.4, *p* = 0.85, Cohen’s *d* < 0.01; interaction: *F*_1,1195_ = 2.75, *p* = 0.10, Cohen’s *d* < 0.01).

### The effect of empathy

Perceived pain intensity was positively and strongly correlated with induced personal distress from the videos (*r* = 0.62, *p* < 0.001), as well as with the perspective taking scale of the IRI (*r* = 0.11, *p* < 0.001). Linear regression of the IRI sub-domains on pain intensity (*F*_3,1995_ = 5.53, *p* = 0.001, *R* = 0.12, constant = 24.0) revealed significant positive predictive effects of perspective taking (*B* = 0.24, *t* = 3.65, *p* < 0.001, *β* = 0.11), and personal distress (*B* = 0.14, *t* = 2.06, *p* = 0.04, *β* = 0.07), but not EC (*B* = −0.02, *t* = −0.31, *p* = 0.76, *β* = −0.01). Induced personal distress correlated positively with perspective taking (*r* = 0.07, *p* = 0.01) and EC (*r* = 0.08, *p* < 0.01) but not with the personal distress subscale of the IRI (*r* = 0.004, *p* = 0.28). Linear regression of the IRI components on induced personal distress (*F*_3,1995_ = 4.56, *p* < 0.01, *R* = 0.11, constant = 12.4) revealed significant positive predictive effects of perspective taking (*B* = 0.14, *t* = 2.07, *p* = 0.04, *β* = 0.06) and EC (*B* = 0.12, *t* = 1.93, *p* = 0.05, *β* = 0.06) and a trend to significance of personal distress (*B* = 0.12, *t* = 1.68, *p* = 0.09, *β* = 0.05).

### The impact on professional quality of life

**Figure 3:**
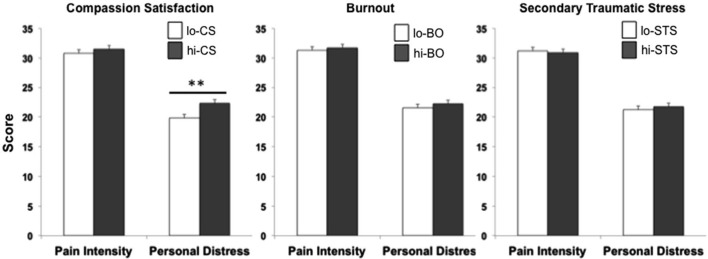
**The effect of professional quality of life**. Comparison of lo- (white bar) and hi- (dark gray bar) participants for the three domains of professional quality of life. A significantly higher induced personal distress was found for hi-CS relative to lo-CS (*p < 0.01). Error bars are *S.E.M*.

**Figure 4:**
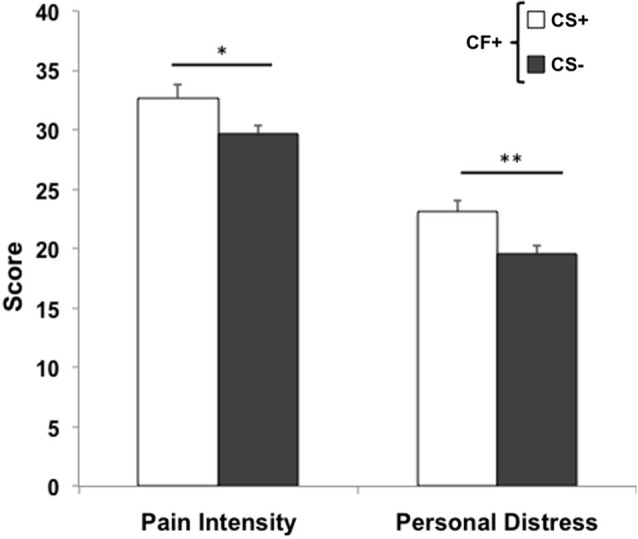
**Compassion Fatigue and Satisfaction**. Within participants who experience compassion fatigue (CF+), those who also experience compassion satisfaction (CS+) perceive targets’ pain more intensely (*p < 0.05) and elicit stronger self-oriented distress (**p = 0.01) than participants who do not experience compassion satisfaction (CS−). Error bars are *S.E.M*.

The only significant correlation found between behavioral measures and domains of professional quality of life was between induced personal distress and compassion satisfaction (*r* = 0.08, *p* < 0.01). Behavioral measures were compared between lo- and hi- groups for each ProQoL domain. While pain intensity ratings were comparable for lo- (*n* = 332) and hi- (*n* = 264) CS participants (*t*_594_ = −1.44, *p* = 0.15), a significant difference was found for induced personal distress (*t*_594_ = −2.89, *p* < 0.01), with hi-CS experiencing significantly higher rates of induced personal distress than lo-CS (Figure 3, left). No significant differences were found for either perceived pain intensity (*t*_651_ = −0.46, *p* = 0.65) or induced personal distress (*t*_651_ = −0.94, *p* = 0.35) between lo- (*n* = 326) and hi- (*n* = 327) BO participants (Figure 3, center). Lo- (*n* = 257) and hi- (*n* = 310) STS participants did not differ on either perceived pain intensity (*t*_555_ = 0.22, *p* = 0.83) or induced personal distress (*t*_555_ = −0.54, *p* = 0.57; Figure 3, right). In fact, within the group of participants who experience compassion fatigue (i.e., scoring for hi-BO and/or hi-STS groups, *n* = 228), those who also experience compassion satisfaction (i.e., scoring for the hi-CS group) perceive significantly more intense levels of pain in others (*t*_226_ = −2.15, *p* = 0.03) and this elicits stronger self-oriented discomfort (*t*_226_ = −2.49, *p* = 0.01) than those who do not experience compassion satisfaction (Figure 4).

## Discussion

Understanding the feelings, thoughts, and behavioral reactions of other conspecifics in distress has a survival value both in protecting the observer from potential dangers, and in protecting or delivering care to others (Craig, 2009). Empathy is at the core of these processes (Decety and Jackson, 2004; Batson, 2009) and, among physicians, it is a key element necessary to be sufficiently motivated to assist patients (Hojat et al., 2002; Bonvicini et al., 2009; Gleichgerrcht and Decety, 2012). Yet, excessively empathic responses may be costly, leading to burnout, emotional and physical exhaustion and professional self-devaluation (Hodges and Biswas-Diener, 2007; Figley, 2012; Gleichgerrcht and Decety, 2013). The present study was designed to understand how different aspects of physicians’ demographic and professional profile and their individual empathic dispositions modulate pain perception and its resulting personal distress, and how these relate to professional quality of life.

We found that while physicians with less experience perceived the pain of others more intensely than more experienced doctors, pain-induced emotional distress was similar irrespective of professional experience. As previously reported (Gleichgerrcht and Decety, 2013), practicing medicine for a longer time does not seem to give physicians a *direct* advantage in learning how to down-regulate the costs of empathy (i.e., compassion fatigue). Instead, the present findings suggest that professional experience may help them desensitize more specifically against the pain of others (Neumann et al., 2011; Haque and Waytz, 2012). In other words, medical experience contributes to the down-regulation of EC but not personal distress. This further supports previous studies showing that expertise leads to decreased sensitivity to the pain of others as reflected by attenuated neuro-hemodynamic activity in the pain matrix (ACC, insula and PAG) combined with an increased activation of prefrontal networks involved in cognitive control (Cheng et al., 2007; Decety et al., 2010b). Because EC has been found to be associated with compassion satisfaction and personal distress linked with compassion fatigue (Gleichgerrcht and Decety, 2013), there could be a potential detrimental effect of professional experience in the absence of other mechanisms aimed at regulating empathy; that is, prolonged exposure to the pain of others alone may potentially decrease empathic concern, and thus it may also decrease the positive aspects of professional quality of life. Moreover, because a number of studies have demonstrated that emotion sharing plays a critical role to elicit helping behavior (Batson et al., 2004; Batson, 2006; Pavey et al., 2012), subjective ratings of perceived pain intensity should be at a level high enough so as to surpass the threshold that leads to an empathic response. Converging results from this study and previous reports therefore suggest that a certain level of other-oriented empathy is necessary to help others and to make that helping experience a positive one for physicians professional life, all of which could be attenuated by the effect of experience. For this reason, longitudinal monitoring of empathy and empathy-related attitudes and healthcare practices (e.g., time spent with the patient, communication effectiveness, eye contact, etc.) is essential for older and more experienced physicians.

Based on our findings, it seems that how much pain physicians perceive in their patients is related to their ability to put themselves in their shoes (perspective taking component of empathy), as well as their proneness to experience personal distress. In fact, the discomfort induced by a target of empathy (as measured by the behavioral task with patient videos) was related to an individual tendency to elicit feelings of worriedness for the patient (empathic concern).

Our study also revealed that the sensitivity to the pain of others is not the same for all patients. When considering their gender, the discomfort or suffering of female patients seems to be perceived as more intense than that of male patients, independently of the physician’s own gender, but especially so if the physician is a woman. Most previous research has focused on differences in empathy of the perceiver, rather than the agent eliciting the empathic response. Although not without controversy, results in the general population tend to show higher empathic sensitivity ratings among females (e.g., Lennon and Eisenberg, 1987; Jaffee and Hyde, 2000; Baron-Cohen et al., 2001; Mestre et al., 2009). For physicians, both self-reports (e.g., Hojat et al., 2002, 2009; Gleichgerrcht and Decety, 2013) and clinical attitudes related to compassion (Bertakis et al., 1995; Roter et al., 2002) have also revealed enhanced empathy among females. Our results show, nonetheless, that at the behavioral level, the gender of the observer does not influence pain sensitivity (i.e., male and female doctors report similar amounts of pain for the video-clips). On the contrary, what may modulate an empathic effect is whose pain we perceive, as female examinees elicited stronger levels of pain perception. This finding is in accordance with previous reports showing that the gender of the target of empathy can impact pain perception, which in turn can be further affected by individual dispositions and contexts (Coll et al., 2012).

It is thus possible that the difference in *subjective* ratings of empathy in males and females does not actually translate into behavioral responses in physicians of either gender. This divergence between self-reports and behavioral (e.g., Derntl et al., 2010) and neurophysiological (e.g., Michalska et al., 2013) measures of empathy has been previously observed in the general population.

The findings of the effect of patient gender on physician’s perception of pain are relevant for care management, as they can influence diagnostic and therapeutic decisions. For example, if a female physician is assessing a male patient’s abdominal pain, she may misleadingly underestimate his suffering and neglect a case of acute abdomen. In fact, previous findings (Verbrugge and Steiner, 1981) have revealed that, even after controlling for relevant clinical factors such as chief complaint, severity, and diagnosis, among others, female patients receive more total prescriptions and return appointments than male patients. It is thus important to make physicians aware of findings of this caliber, as unconscious biases may undermine or exaggerate their perception of patients’ suffering. This also calls for further research on the impact of patients’ gender in physicians’ attitudes towards pain, for example, by combining actual reports of the pain experienced by patients of each gender and the level of pain perceived by male and female doctors differentially (e.g., see Wager et al., 2013).

This study also contributes to the idea that a minimum level of empathy is necessary to benefit from the positive aspects of professional quality of life. Participants experienced compassion satisfaction when they reported a certain level of personal distress in response to someone’s suffering. One likely explanation for this surprising finding is that enhanced empathic responses result in increased motivation to help those in distress, thus promoting a more satisfactory experience for the health professional. Further evidence to support this claim is that specialty fields identified as highly demanding in terms of emotional exhaustion were associated with increased target-induced (i.e., behavioral) personal distress but not dispositional personal distress, suggesting perhaps that it is more a consequence of the field in which they practice medicine than a causal factor influencing their choice of specialty. This is in line with other studies showing that the highest scores of empathy exist among physicians in fields at the front line of primary care even in the presence of very high rates of burnout (for review, see Newton, 2013). At the intersection between empathy and professional quality of life are most likely the complex processes of emotion regulation and cognitive control. Being able to elicit the appropriate response (both qualitatively and quantitatively) in the right social context (e.g., operating room vs. bedside follow-up) is obviously a challenging capacity resulting from numerous factors, some of which were investigated in the present study.

Some caveats in our experimental set up call for further research. First, pain intensity was only rated from clips showing the facial expression of patients. Pain can be exhibited physically in several other ways (e.g., upper body bent forward, hand pressing against abdomen, etc.), reason why future studies should use whole-body stimuli to test the association between these variables. It would be valuable to look at alternative ways of classifying medical specialties to determine whether the effects found in the present study hold after using other criteria; for example, by gathering objective data relating to fatigue, such as the number of on-call nights, the number of patients seen daily, and similar measures. Alternatively, respondents could report their perceptions of their own fields in terms of emotional demand to determine whether converging trends are found with the present findings that would further support the rather subjective, third-party classification employed here. We also encourage gathering “ecological” measures in healthcare settings, such as degree of pain experienced by patients and variables tapping on the intensity of physician-patient interactions. These kinds of measures will be essential in clarifying how experimental results translate to real-life settings. Another interesting measure worthy of further research is whether physicians’ familiarity with pain influences their pain perception and compassion fatigue. For instance, asking participants to determine their monthly volume of patients and the proportion of their patient population experiencing pain.

## Conclusion

While, everybody agrees that empathy is an integral component of effective medical care, improving patient outcomes and increasing physician satisfaction, the relationship between individual dispositions, empathy (and its components), and professional quality of life is not simple. Understanding the specific association between different facets of empathy and professional quality of life can lead to the design of evidence-based training programs for health professionals and medical students to help them down-regulate the appropriate aspects of empathy so as to avoid burnout and compassion fatigue, while ensuring that they are still capable to eliciting an empathic response strong enough to be motivated to assist patients as needed.

## Conflict of interest statement

The authors declare that the research was conducted in the absence of any commercial or financial relationships that could be construed as a potential conflict of interest.

## References

[B1] Baron-Cohen S. Wheelwright S. Hill J. Raste Y. Plumb I. (2001). The “Reading the mind in the eyes” test revised version: a study with normal adults and adults with Asperger syndrome or high-functioning autism. J. Child Psychol. Psychiatry. 57, 241–251 10.1111/1469-7610.00715 11280420

[B2] Batson C. D. (2006). “‘Not all is self-interest after all’: economics of empathy-induced altruism,” in Social Psychology and Economics, eds De Cremer D. Zeelenberg M. Murnighan J. K. (Mahwah, NJ: Lawrence Erlbaum Associates), 281–299

[B3] Batson C. D. Ahmad N. Stocks E. L. (2004). “Benefits and liabilities of empathy-induced altruism,” in The Social Psychology of Good and Evil, ed Miller A. G. (New York: Guilford Press), 359–385

[B4] Batson D. C. (2009). “These things called empathy: eight related but distinct phenomena,” in The Social Neuroscience of Empathy, eds Decety J. Ickes W. J. (Cambridge, MA: The MIT Press), 3–15

[B5] Benbassat J. Baumal R. (2004). What is empathy and how can it be promoted during clinical clerkships? Acad. Med. 79, 832–839 10.1097/00001888-200409000-00004 15326005

[B6] Bertakis K. D. Helms L. J. Callahan E. J. Azari R. Robbins J. A. (1995). The influence of gender on physician practice style. Med. Care 33, 407–416 10.1097/00005650-199504000-00007 7731281

[B7] Bonvicini K. A. Perlin M. J. Bylund C. L. Carroll G. Rouse R. A. Goldstein M. G. (2009). Impact of communication training on physician expression of empathy in patient encounters. Patient Educ. Couns. 75, 3–10 10.1016/j.pec.2008.09.007 19081704

[B8] Burns D. D. Nolen-Hoeksema S. (1992). Therapeutic empathy and recovery from depression in cognitive-behavioral therapy: a structural equation model. J. Consult. Clin. Psychol. 60, 441–449 10.1037//0022-006x.60.3.441 1619098

[B9] Cheng Y. Chen C. Y. Lin C. P. Chou K. H. Decety J. (2010). Love hurts: an fMRI study. Neuroimage 51, 923–929 10.1016/j.neuroimage.2010.02.047 20188182

[B10] Cheng Y. Lin C. P. Liu H. L. Hsu Y. Y. Lim K. E. Hung D. (2007). Expertise modulates the perception of pain in others. Curr. Biol. 17, 1708–1713 10.1016/j.cub.2007.09.020 17900903

[B11] Coll M.-P. Budell L. Rainville P. Decety J. Jackson P. L. (2012). The role of gender in the interaction between self-pain and the perception of pain in others. J. Pain 13, 695–703 10.1016/j.jpain.2012.04.009 22705065

[B12] Craig K. D. (2004). Social communication of pain enhances protective functions. Pain 107, 5–6 10.1016/s0304-3959(03)00264-1 14715382

[B13] Craig K. D. (2009). The social communication model of pain. Can. Psychol. 50, 22–32 10.1037/a0014772

[B14] Craig K. D. Patrick C. J. (1985). Facial expression during induced pain. J. Pers. Soc. Psychol. 48, 1080–1091 10.1037//0022-3514.48.4.1089 3989673

[B15] Davis M. H. (1983). Measuring individual differences in empathy: evidence for a multidimensional approach. J. Pers. Soc. Psychol. 44, 113–126 10.1037/0022-3514.44.1.113

[B16] Decety J. (2009). Empathy, sympathy and the perception of pain. Pain 145, 365–366 10.1016/j.pain.2009.08.006 19716658

[B58] Decety J. (2010). The neurodevelopment of empathy in humans. Dev. Neurosci. 32, 257–267 10.1159/000317771 20805682 PMC3021497

[B17] Decety J. (2011). Dissecting the neural mechanisms mediating empathy. Emotion Rev. 3, 92–108 10.1177/1754073910374662

[B18] Decety J. Jackson P. L. (2004). The functional architecture of human empathy. Behav. Cogn. Neurosci. Rev. 3, 71–100 10.1177/1534582304267187 15537986

[B19] Decety J. Svetlova M. (2012). Putting together phylogenetic and ontogenetic perspectives on empathy. Dev. Cogn. Neurosci. 2, 1–24 10.1016/j.dcn.2011.05.003 22682726 PMC6987713

[B20] Decety J. Echols S. Correll J. (2010a). The blame game: the effect of responsibility and social stigma on empathy for pain. J. Cogn. Neurosci. 22, 985–997 10.1162/jocn.2009.21266 19425830

[B21] Decety J. Norman G. J. Berntson G. G. Cacioppo J. T. (2012). A neurobehavioral evolutionary perspective on the mechanisms underlying empathy. Prog. Neurobiol. 98, 38–48 10.1016/j.pneurobio.2012.05.001 22580447

[B22] Decety J. Yang C. Y. Cheng Y. (2010b). Physicians down-regulate their pain empathy response: an event-related brain potential study. Neuroimage 50, 1676–1682 10.1016/j.neuroimage.2010.01.025 20080194

[B23] Del Canale S. Louis D. Z. Maio V. Wang X. Rossi G. Hojat M. (2012). The relationship between physician empathy and disease complications: an empirical study of primary care physicians and their diabetic patients in Parma, Italy. Acad. Med. 87, 1243–1249 10.1097/acm.0b013e3182628fbf 22836852

[B24] Derntl B. Finkelmeyer A. Eickhoff S. Kellermann T. Falkenberg D. I. Schneider F. (2010). Multidimensional assessment of empathic abilities: neural correlates and gender differences. Psychoneuroendocrinology 35, 67–82 10.1016/j.psyneuen.2009.10.006 19914001

[B25] Echols S. Correll J. (2012). “It’s more than skin deep: empathy and helping behavior across social groups,” in Empathy: From Bench to Bedside, ed Decety J. (Cambridge MA: MIT Press), 55–71

[B26] Eisenberger N. (2011). “Why rejection hurts: what social neuroscience has revealed about the brain’s response to social rejection,” in Empathy: From Bench to Bedside, eds Decety J. Cacioppo J. T. (New York: Oxford University Press), 586–598

[B27] Feighny K. M. Monaco M. Arnold L. (1995). Empathy training to improve physician-patient communication skills. Acad. Med. 70, 435–436 10.1097/00001888-199505000-00031 7748400

[B28] Figley C. R. (2012). “The empathic response in clinical practice: antecedents and consequences,” in Empathy: From Bench to Bedside, ed Decety J. (Cambridge MA: MIT Press), 263–273

[B29] Gleichgerrcht E. Decety J. (2012). “The costs of empathy among health professionals,” in Empathy: From Bench to Bedside, ed Decety J. (Cambridge: MIT Press), 245–2611

[B30] Gleichgerrcht E. Decety J. (2013). Empathy in clinical practice: how individual dispositions, gender and experience moderate empathic concern, burnout and emotional distress in physicians. PLoS One 8:e61526 10.1371/journal.pone.0061526 23620760 PMC3631218

[B31] Halpern J. (2012). “Clinical empathy in medical care,” in Empathy: From Bench to Bedside, ed Decety J. (Cambridge: MIT Press), 229–244

[B32] Handford C. Lemon J. Grimm M. C. Vollmer-Conna U. (2013). Empathy as a function of clinical exposure—reading emotion in the eyes. PLoS One 8:e65159 10.1371/journal.pone.0065159 23755185 PMC3673998

[B33] Haque O. S. Waytz A. (2012). Dehumanization in medicine: causes, solutions and functions. Perspect. Psychol. Sci. 7, 176–186 10.1177/1745691611429706 26168442

[B34] Hodges S. D. Biswas-Diener R. (2007). “Balancing the empathy expense account: strategies for regulating empathic response,” in Empathy in Mental Illness, eds Farrow T. F. D. Woodruff P. W. R. (Cambridge: Cambridge Uniersity Press), 389–405

[B35] Hojat M. Gonnella J. S. Nasca T. J. Mangione S. Vergare M. Magee M. (2002). Physician empathy: definition, components, measurement and relationship to gender and specialty. Am. J. Psychiatry 159, 1563–1569 10.1176/appi.ajp.159.9.1563 12202278

[B36] Hojat M. Louis D. Z. Markham F. W. Wender R. Rabinowitz C. Gonnella J. S. (2011). Physicians’ empathy and clinical outcomes for diabetic patients. Acad. Med. 86, 359–364 10.1787/276757872478 21248604

[B37] Hojat M. Vergare M. J. Maxwell K. Brainard G. Herrine S. K. Isenberg G. A. (2009). The devil is in the third year: a longitudinal study of erosion of empathy in medical school. Acad. Med. 84, 1182–1191 10.1097/acm.0b013e3181b17e55 19707055

[B38] Jaffee S. Hyde J. S. (2000). Gender differences in moral orientation: a meta-analysis. Psychol. Bull. 126, 703–726 10.1037//0033-2909.126.5.703 10989620

[B39] Lamm C. Batson C. D. Decety J. (2007). The neural substrate of human empathy: effects of perspective-taking and cognitive appraisal. J. Cogn. Neurosci. 19, 42–58 10.1162/jocn.2007.19.1.42 17214562

[B40] Lamm C. Decety J. Singer T. (2011). Meta-analytic evidence for common and distinct neural networks associated with directly experienced pain and empathy for pain. Neuroimage 54, 2492–2502 10.1016/j.neuroimage.2010.10.014 20946964

[B41] Lennon R. Eisenberg N. (1987). “Gender and age differences in empathy and sympathy,” in Empathy and its Development, eds Eisenberg N. Strayer J. (Cambridge, UK: Cambridge Uniersity Press), 195–217

[B42] Mercer S. W. Reynolds W. J. (2002). Empathy and quality of care. Br. J. Gen. Pract. 52, S9–S12 10.1007/0-387-33608-7 12389763 PMC1316134

[B43] Mestre M. V. Samper P. Frías M. D. Tur A. M. (2009). Are women more empathetic than men? A longitudinal study in adolescence. Span. J. Psychol. 12, 76–83 Available at: http://www.ncbi.nlm.nih.gov/pubmed/19476221 [Accessed July 13, 2012]. 10.1017/s1138741600001499 19476221

[B44] Michalska K. J. Kinzler K. D. Decety J. (2013). Age-related sex differences in explicit measures of empathy do not predict brain responses across childhood and adolescence. Dev. Cogn. Neurosci. 3, 22–32 10.1016/j.dcn.2012.08.001 23245217 PMC6987715

[B45] Morse D. S. Edwardsen E. Gordon H. S. (2008). Missed opportunities for interval empathy in lung cancer communication. Arch. Intern. Med. 168, 1853–1858 10.3410/f.1123346.584149 18809811 PMC2678758

[B46] Neumann M. Edelhäuser F. Tauschel D. Fischer M. R. Wirtz M. Woopen C. (2011). Empathy decline and its reasons: a systematic review of studies with medical students and residents. Acad. Med. 86, 996–1009 10.1097/acm.0b013e318221e615 21670661

[B47] Newton B. W. (2013). Walking a fine line: is it possible to remain an empathic physician and have a hardened heart? Front. Hum. Neurosci. 7:233 10.3389/fnhum.2013.00233 23781181 PMC3678078

[B48] Pavey L. Greitemeyer T. Sparks P. (2012). “I help because I want to, not because you tell me to”: empathy increases autonomously motivated helping. Pers. Soc. Psychol. Bull. 38, 681–689 10.1177/0146167211435940 22326945

[B49] Rakel D. P. Hoeft T. J. Barrett B. P. Chewning B. A. Craig B. M. Niu M. (2009). Practitioner empathy and the duration of the common cold. Fam. Med. 41, 494–501 10.1016/j.bbi.2008.04.014 19582635 PMC2720820

[B50] Rakel D. Barrett B. Zhang Z. Hoeft T. Chewning B. Marchand L. (2011). Perception of empathy in the therapeutic encounter: effects on the common cold. Patient Educ. Couns. 85, 390–397 10.1016/j.pec.2011.01.009 21300514 PMC3107395

[B51] Riess H. (2010). Empathy in medicine–a neurobiological perspective. JAMA 304, 1604–1605 10.1001/jama.2010.1455 20940387

[B52] Roter D. L. Hall J. A. Aoki Y. (2002). Physician gender effects in medical communication. JAMA 288:756 10.1001/jama.288.6.756 12169083

[B53] Schattner A. (2012). Who cares for empathy? QJM 105, 287–290 10.4016/34345.01 22240392

[B54] Stamm B. H. (2008). The ProQOL Test Manual. 2nd Edn. Baltimore: Sidran Press

[B55] van Baaren R. Decety J. Dijksterhuis A. van de Leij A. van Leeuwenand M. L. (2009). “Being imitated: consequences of nonconsciously showing empathy,” in The Social Neuroscience of Empathy, eds Eisenberg J. Decety J. (Cambridge: MIT Press), 31–42

[B56] Verbrugge L. M. Steiner R. P. (1981). Physician treatment of men and women patients: sex bias or appropriate care? Med. Care 19, 609–632 10.1097/00005650-198106000-00005 7266112

[B57] Wager T. D. Atlas L. Y. Lindquist M. A. Roy M. Woo C.-W. Kross E. (2013). An fMRI-based neurologic signature of physical pain. N. Engl. J. Med. 368, 1388–1397 10.1056/NEJMoa1204471 23574118 PMC3691100

